# Can yeast isolation be predicted in complicated secondary non-postoperative intra-abdominal infections?

**DOI:** 10.1186/s13054-015-0790-3

**Published:** 2015-02-27

**Authors:** Hervé Dupont, Mathieu Guilbart, Alexandre Ntouba, Mélanie Perquin, Sandra Petiot, Jean-Marc Regimbeau, Taieb Chouaki, Yazine Mahjoub, Elie Zogheib

**Affiliations:** Department of Anesthesiology and Critical Care Medicine, Amiens University Medical Center, 80054 Amiens, Cedex 1 France; INSERM U1088, Jules Verne University of Picardy, Chemin du Thil, 80039 Amiens, Cedex 1 France; Digestive and Metabolic Surgery Department, Amiens University Medical Center, 80054 Amiens, Cedex 1 France; Mycology Laboratory, Amiens University Medical Center, 80054 Amiens, Cedex 1 France

## Abstract

**Introduction:**

The aim of this study was to create a predictive score for yeast isolation in patients with complicated non-postoperative intra-abdominal infections (CNPIAI) and to evaluate the impact of yeast isolation on outcome.

**Methods:**

All patients with a CNPIAI undergoing emergency surgery over a three-year period were included in the retrospective cohort (RC, n = 290). Patients with a yeast-positive peritoneal fluid culture (YP) were compared with patients with a yeast-negative culture (YN). Multivariate logistic regression was used to identify factors independently associated with yeast isolation and a predictive score was built. The score’s performance was then established in the prospective cohort (PC, n = 152) over an 18-month period. Outcome of the whole cohort was evaluated and independent risks factors of mortality searched.

**Results:**

In the RC, 39 patients (13.4%) were YP. Four factors were independently associated with the YP group: length of stay before surgery ≥48 h (odds ratio (OR) (95% confidence interval (CI)) = 3.1 (1.4 to 6.9), *P* = 0.004, 1 point), per-operative cardiovascular failure (2.4 (1.1 to 5.8), *P* = 0.04, 1 point), generalized peritonitis (6.8 (2.7 to 16.7), *P* <0.001, 2 points) and upper gastrointestinal tract perforation (2.5 (1.2 to 5.6), *P* = 0.02, 1 point). In the PC, the area under the curve (95%CI) of the predictive score’s receiver operating characteristic curve was 0.79 (0.72 to 0.86). For predicting an intra-abdominal candidiasis (IAC), a score ≥3 had a sensitivity of 0.60, a specificity of 0.84, a positive predictive value of 0.49 and a negative predictive value of 0.89. Furthermore, yeast isolation was associated with worse outcome and independently associated with mortality in the whole cohort (OR = 2.15; 95%CI (1.03 to 4.46), *P* = 0.04).

**Conclusions:**

The new predictive score can be used to rule out intra-abdominal candidiasis and thus avoid the initiation of antifungal treatment. It is suited to less severe infections than previously published scores. IAC is associated independently with an increased mortality in this population.

## Introduction

The pathogenicity of yeasts isolated in the peritoneal fluid of patients with complicated intra-abdominal infections (and especially community-acquired infections (CAIs)) is subject to debate. There are some data to suggest that yeasts have an impact on the outcome in cases of peptic ulcer perforation [1]. Non-postoperative nosocomial intra-abdominal infections (NPNIAIs) share certain microbiological characteristics with CAIs [2], such as the frequency of yeast isolation in peritoneal fluid samples. In view of the high mortality rate observed in patients with post-operative infections [3] and patients with organ failure admitted to the intensive care unit (ICU) [4], the pathogenicity of yeast in these contexts has been investigated more. However, the guidelines on the management of these types of infection are essentially based on extrapolation of data on candidemia [5,6]. Even though yeast intra-abdominal infections can be candidemic [7], the frequency is low and the diseases do not have the same course [8]. A number of scores have been developed in order to predict the occurrence of candidemia in high-risk patients, including the colonization index [9], Leon *et al*.’s *Candida* score [10] and a clinical rule [11]. However, none of these scores is suitable for complicated intra-abdominal infections. Furthermore, each of these scores has a high negative predictive value (NPV, for ruling out yeast infection) rather than a high positive predictive value (PPV, for initiating treatment) [12]. Ten years ago, Dupont *et al*. developed a score for severe complicated intra-abdominal infections in the ICU [13]. This is still the only available score with a moderately good PPV and NPV [13]. However, it was developed in a severe population of ICU patients. There are few data on less severe patients having undergone emergency surgery. The objectives of the present study were to (i) build a predictive score for yeast isolation in the peritoneal fluid of patients with complicated non-postoperative intra-abdominal infections (CNPIAI) in a retrospective cohort of patients and (ii) validate the score in a separate prospective cohort. The new score will be compared with previously described scores. The relationship between intra-abdominal candidiasis (IAC) and the outcomes for patients with complicated CNPNIAIs was also evaluated.

## Material and methods

### Study design and patients

This study was conducted in three parts. In the first part (score construction), patients with a CNPIAI and having undergone emergency surgery in our tertiary university hospital were retrospectively included over a three-year period (from January 2009 to December 2011). Patients with yeast-positive (YP) peritoneal fluid culture were compared with patient with a yeast-negative (YN) culture. A predictive score was built by taking account of factors independently associated with IAC in the cohort. In the second part of the study (score validation), patients were included in a prospective cohort over an 18-month period (from January 2012 to June 2013). The performance of the new score was compared with previously published predictive scores (Dupont *et al*. [13], Leon *at al*. [10] and Paphitou *et al*. [14]). In the third part, the impact of IAC on outcome in the whole cohort was assessed.

The study’s objectives and procedures were approved by the local investigational review board (Commission d’Evaluation Ethique de la Recherche Non Interventionnelle, Amiens, France). In accordance with French legislation, the need for informed consent was waived because of the observational nature of the study.

Patients with infected acute pancreatitis, postoperative nosocomial infections, acute trauma perforation <6 hours and primary peritonitis (such as infected ascites) were not included in the study.

### Surgery and microbiological management

Surgery was performed by an experienced, trained team in accordance with the above-mentioned guidelines for the treatment of complicated intra-abdominal infections [15]. The definition of complicated intra-abdominal infection used is that presented in the Infectious Diseases Society of America (IDSA) guidelines: ‘Complicated intra-abdominal infection extends beyond the hollow viscus of origin into the peritoneal space and is associated with either abscess formation or peritonitis’ [6]. Either laparoscopy or laparotomy was performed (depending on the diagnosis and the surgeon’s choice). All peritoneal fluid samples were sent for microbiological and mycological testing. Antimicrobial therapy was initiated as soon as possible and in accordance with local treatment protocols. Treatment of yeast infection was only considered if the patient had organ failure. At least one preoperative blood culture was available for each patient. The mycology department performed yeast cultures and susceptibility testing. A strain of *Candida* was considered resistant to fluconazole for a minimum inhibitory concentration >32 μg.ml^−1^.

### Definitions and data collection

The etiology of the intra-abdominal infection, the extent of the infection (generalized or localized) and the perforation site were recorded. The hospital length of stay before surgery was noted. Demographic data and the underlying disease were noted from the patient’s medical records. A number of severity scores were calculated: the American Society of Anesthesiologist score [16], the Mannheim peritonitis index [17], the Acute Physiology and Chronic Health Evaluation II (APACHE II) score [18], the Simplified Acute Physiology Score II (SAPS II) [19] and the Sepsis-related Organ Failure Assessment (SOFA) score [20]. Cardiovascular failure was defined by the need for norepinephrine during surgery (despite fluid challenge). Respiratory failure was defined by the need for more than 24 hours of mechanical ventilation. The ICU admission rate, lengths of stay (in the ICU or a hospital ward) and in-hospital mortality were assessed.

### Statistical analysis

Results are expressed as mean ± standard deviation (SD) or number (proportion). First, patients in the YP group were compared with those in the YN group via a chi-squared test (with Yates’ correction, if necessary) for qualitative variables and a two-sided *t* test for quantitative variables. A multivariate stepwise logistic regression model (backward Wald model) was built in order to identify any independent factors for yeast isolation in patients with intra-abdominal infections. Only significant variables (*P* <0.05) in a univariate analysis were included in the multivariate model. All potential explanatory variables included in the multivariate analyses were subjected to a collinearity analysis in a correlation matrix. Intercorrelated variables were not included in the multivariate model (tolerance <0.3 and variance inflation factor >3). Adjusted odds ratios (ORs) and their 95% confidence intervals (95%CIs) are reported. The constant (intercept) was only included in the model when statistically significant [21]. The Hosmer-Lemeshow test was used to assess the model’s goodness of fit [21]. The statistical significance of individual regression coefficients was assessed with the Wald chi-squared test [21]. The model’s predicted probabilities were validated with the *c* statistic (corresponding to the model’s area under the curve (AUC)) [21].

A score was built according to the ORs in the multivariate analysis and was tested in a receiver operating characteristic (ROC) analysis [22]. The best score’s cutoff was calculated for the best Youden index. The score’s performance was tested in a prospective (validation) cohort, according to the same analysis. The AUC of the ROC curve of new score was compared with the AUC of previously published scores in the same cohort using Hanley and McNeil tests.

A second multivariate analysis was performed to identify the impact of yeast isolation in mortality of the whole cohort. The threshold for statistical significance was set to *P* ≤0.05. Statistical analyses were performed with PASW Statistics 18 software (IBM Inc., Chicago, IL, USA) and MedCalc 12.7.5 software (MedCalc Software, Ostend, Belgium).

## Results

Four hundred and forty-four patients were included in the study (290 in the retrospective cohort and 152 in the prospective cohort). Location of perforation, main etiologies and microbiological cultures of the peritoneal fluid are exposed in Tables 1 and 2. Bacteremia occurred in 9.3% of the patients and no candidemia was observed.

**Table 1:** **Location and etiologies of complicated non-postoperative intra-abdominal infections in the whole cohort of patients**

	**Whole cohort**
	**(n = 442)**
**Lower gastrointestinal tract**	**312 (70.6)**
Appendicitis	133 (30.1)
Diverticulitis	75 (17)
Inflammatory bowel disease	10 (2.3)
Malignancy	18 (4.1)
Ischemic	39 (8.8)
Miscellaneous	37 (8.4)
**Upper intestinal tract**	**130 (29.4)**
Biliary tract	76 (17.2)
Ulcus disease	43 (9.7)
Ischemic	6 (1.4)
Miscellaneous	5 (1.0)

Results are expressed as the number (proportion, in %).

**Table 2:** **Results of the peritoneal fluid cultures in the whole cohort of patients with complicated non-postoperative intra-abdominal infections (n = 442)**

	**Isolates**
	**(n = 927)**
**Aerobes**	**677** (73.0)
***Gram-negative bacilli***	***426*** *(45.9)*
*Escherichia coli*	270
*Klebsiella* spp.	43
*Enterobacter* spp.	12
Non-fermenting bacilli	30
Miscellaneous	71
***Gram-positive cocci***	***251*** *(27.1)*
Streptococci	109
Staphylococci	14
Enterococci	128
**Anaerobes**	**178** (19.2)
*Bacteroides* spp.	138
*Clostridium* spp.	18
Miscellaneous	22
**Fungi** ^*^	**72** (7.7)
*Candida albicans*	47
*Candida glabrata*	8
*Candida tropicalis*	7
*Candida krusei*	3
Miscellaneous	7

Results are expressed as the number (proportion, in %). ^*^Thirteen patients had a pure culture of *Candida* (18.8% of the fungal infections).

### Predictive factors for yeast isolation

Two hundred and ninety patients were included in the retrospective cohort. Of these, 39 (13.4%) had an intra-abdominal candidiasis (72.5% of *Candida albicans*). Fifteen percent of the strains were fluconazole-resistant. The demographic characteristics of the YP and YN groups are summarized in Table 3. There were no significant intergroup differences other than a higher proportion of patients on immunosuppression in the YP group (*P* = 0.006). In all, 74.1% of the patients had a CAI; these were essentially gallbladder perforations (n = 56, 19.3%), appendix perforations (n = 90, 31.1%) and colon perforations (n = 81, 27.9%). There were marked intergroup differences in the severity scores and types of infection (Table 3). Patients in the YP group were significantly more likely to have worse severity scores (*P* <0.001 for all), NPNIAIs (*P* <0.001), generalized infections (<0.001), upper gastrointestinal tract perforation (*P* <0.001) and ongoing antimicrobial therapy (≥48 hours) (*P* = 0.01). The results of the multivariate analysis are reported in Table 4. Four independent factors were predictive of IAC: length of stay before surgery ≥48 h, per-operative cardiovascular failure, generalized peritonitis and upper gastrointestinal tract perforation. The model’s Wald chi-squared statistic was 50.2 (df = 5, *P* <0.001). The Hosmer-Lemeshow test statistic was 9.69 (df = 6, *P* = 0.14). The model’s *c* statistic was 0.83 (0.77 to 0.89).

**Table 3:** **Demographic data for the retrospective cohort according of the presence (yeast positive) or absence (yeast negative) of yeast in the peritoneal fluid culture**

	**Yeast positive**	**Yeast negative**	***P*** **value**
	**(n = 39)**	**(n = 251)**	
Age	65 ± 18	59 ± 22	0.08
Female gender	20 (51.3)	125 (49.8)	0.98
BMI (kg.m^−2^)	26.6 ± 7.2	25.9 ± 5.7	0.95
Underlying diseases			
Prior abdominal surgery	7 (17.9)	46 (18.1)	0.87
Diabetes	7 (17.9)	40 (15.9)	0.93
Immunosuppression	7 (17.9)	12 (4.8)	0.006
Chronic cardiovascular disease	22 (56.4)	120 (47.8)	0.41
Chronic renal failure	3 (7.7)	9 (3.6)	0.15
ASA status	2.9 ± 0.6	2.5 ± 0.8	0.001
Mannheim peritonitis index score	22.9 ± 7.6	16.0 ± 8.1	<0.001
APACHE II score	14.6 ± 10.9	8.0 ± 7.9	<0.001
SAPS II score	36.2 ± 20.6	24.7 ± 15.2	<0.001
SOFA score	4.9 ± 6.5	1.9 ± 3	<0.001
Type of infection			
Community-acquired	19 (48.7)	196 (78.1)	<0.001
LOS ≥48 h before surgery	20 (51.3)	55 (21.9)	
Generalized peritonitis	31 (79.5)	95 (37.8)	<0.001
Upper gastrointestinal tract location	18 (46.2)	74 (29.6)	<0.001
Ongoing AB ≥48 h	12 (30.8)	35 (13.9)	0.01

Results are expressed as the mean ± standard deviation or the number (proportion, in %). BMI, body mass index; ASA, American Society of Anesthesiology; APACHE II, Acute Physiology and Chronic Health Evaluation II; SAPS II, Simplified Acute Physiology Score II; SOFA, Sepsis-related Organ Failure Assessment; LOS, length of stay; AB, antimicrobial therapy.

**Table 4:** **Multivariate analysis of factors independently associated with an intra-abdominal candidiasis in the retrospective cohort with CAIs and NPNIAIs**

**Parameters**	**Adjusted OR**	**95%CI**	***P*** **value**
Per-operative cardiovascular failure	2.43	1.01 - 5.81	0.04
Upper gastrointestinal tract perforation	2.53	1.15 - 5.55	0.02
LOS ≥48 h before surgery	3.15	1.45 - 6.89	0.004
Generalized peritonitis	6.78	2.75 - 16.68	<0.001

CAIs, community-acquired infections; NPNIAIs, non-postoperative nosocomial intra-abdominal infections; OR, odds ratio; CI, confidence interval; LOS, length of stay.

### Construction and validation of the score

A predictive score was built according to the ORs in the multivariate analysis (Table 5). In the retrospective cohort, the area (95%CI) under the curve was 0.82 (0.73 to 0.90), yielding the following characteristics for a score ≥3: sensitivity = 0.75; specificity = 0.77; PPV = 0.34; NPV = 0.95; positive likelihood ratio = 3.2; negative likelihood ratio = 0.34.

**Table 5:** **Predictive score for intra-abdominal candidiasis in complicated non-postoperative intra-abdominal infections**

**Item**	**Value**
LOS ≥48 h before surgery	1 point
Per-operative cardiovascular failure	1 point
Generalized peritonitis	2 points
Upper gastrointestinal tract perforation	1 point

LOS, length of stay.

**Figure 1:**
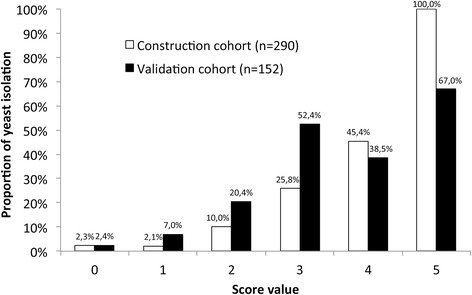
**Comparison of the prevalence of a yeast-positive (YP) peritoneal fluid culture in the retrospective construction cohort (n = 290) and in the prospective validation cohort (n = 152), as a function of the score.**

**Figure 2:**
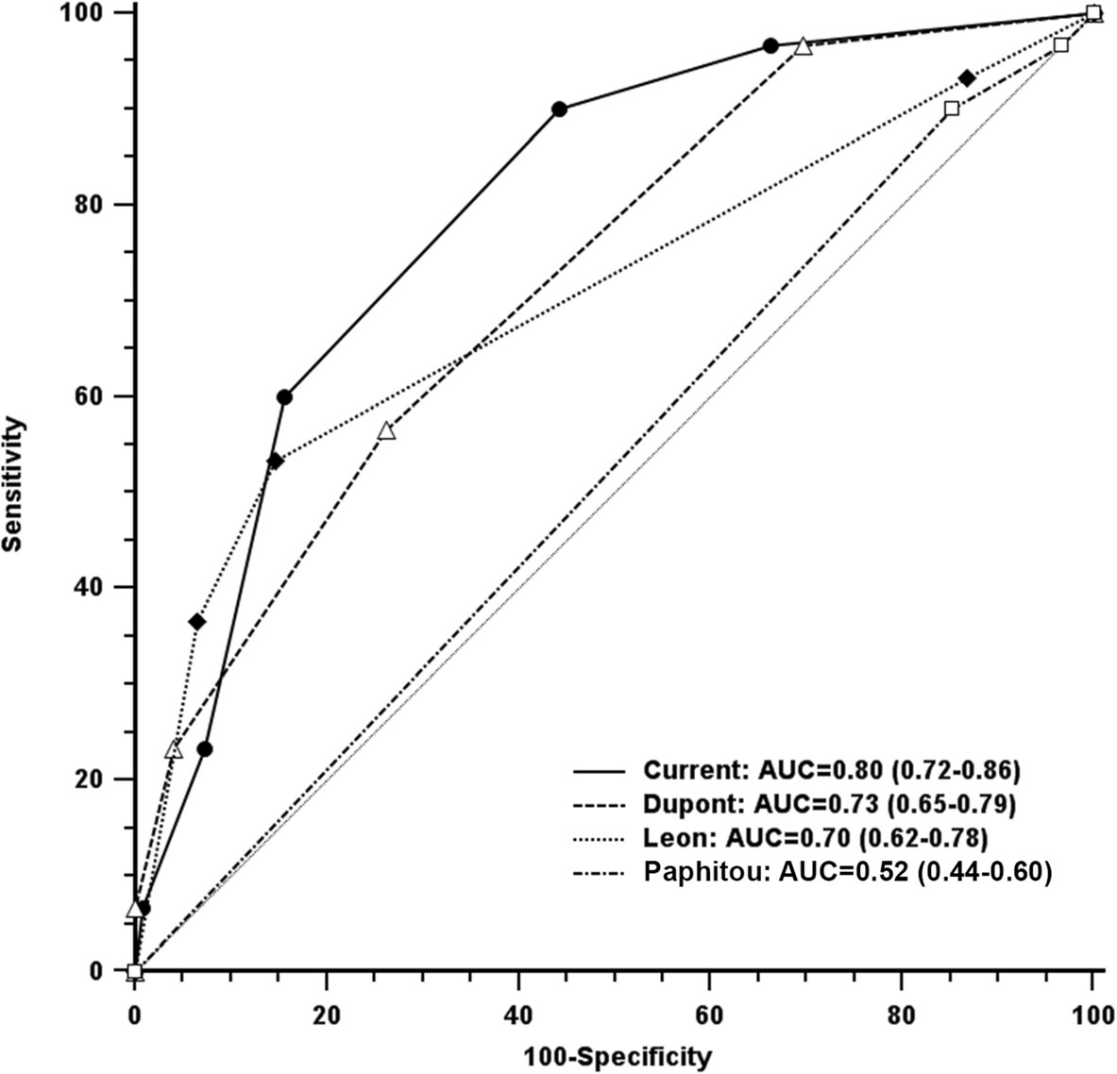
**Comparison of receiver operating characteristic curves between the new score and previously published one in the prospective cohort (Dupont** ***et al*** **.** [13]**, Leon** ***et al*** **.** [10]**, Paphitou** ***et al*** **.** [14]**).**

The score was validated in a prospective cohort of 152 patients. There were no significant demographic or clinical differences between the retrospective and prospective cohorts (Table 6), except for a slightly higher SOFA score in the prospective cohort (*P* = 0.03). Thirty-two strains of yeasts were isolated, with essentially the same distribution as in the retrospective cohort (*C. albicans*: 62.5%) and the same proportion of fluconazole resistance (15.6%). The proportion of YP samples as a function of the score is shown for each cohort in Figure 1. The score’s AUC of the ROC curve for the prospective cohort was 0.79 (0.72 to 0.86). For a predictive score ≥3, the sensitivity was 0.60, the specificity was 0.84, the PPV was 0.49, the NPV was 0.90, the positive likelihood ratio was 3.85 and the negative likelihood ratio was 0.47. The AUC of the ROC curve of the new score is presented in comparison with previously published scores of Dupont *et al*. [13], Leon *et al*. [10] and Paphitou *et al*. [14] in Figure 2. The new score had the best AUC when compared with other scores. However, no statistical differences were observed between the new score and Dupont’s score (*P* = 0.15), the new score and Leon’s score (*P* = 0.17). The former three scores were all significantly better than Paphitou’s score (*P* = 0.0001; *P* = 0.006; *P* = 0.02, respectively).

**Table 6:** **Comparison of the construction and validation cohorts**

	**Construction cohort**	**Validation cohort**	***P*** **value**
	**(n = 290)**	**(n = 152)**	
Age	60.2 ± 21.7	59.4 ± 27.8	0.74
Female gender	145 (50.0)	80 (52.6)	0.62
BMI (kg.m^−2^)	26.0 ± 5.9	26.5 ± 16.8	0.72
Mannheim peritonitis index score	16.9 ± 8.4	17.8 ± 4.2	0.13
SOFA score	2.0 ± 3.9	2.5 ± 1.4	0.03
APACHE II score	8.9 ± 8.6	9.3 ± 7.1	0.57
IAC	39 (13.4)	30 (19.7)	0.10
Community-acquired infection	215 (74.1)	114 (75.0)	0.91
ICU admission	93 (32.1)	57 (37.5)	0.29
Mortality	30 (10.3)	20 (13.2)	0.43

Results are expressed as the mean ± standard deviation or the number (proportion, in %). BMI, body mass index; SOFA, Sepsis-related Organ Failure Assessment; APACHE II, Acute Physiology and Chronic Health Evaluation II; IAC, intra-abdominal candidiasis; ICU, intensive care unit.

### Outcome of the whole cohort

Main outcomes when comparing YP and YN groups are presented in Table 7. Briefly, patients with an IAC had more complications, had undergone more relaparotomies, and had more organ failure requiring ICU admission. Their hospital length of stay was twice as high and mortality three times higher (27.5% vs. 8.3%, *P* <0.001). Univariate and multivariate analysis of mortality is exposed in Table 8. Four independent factors of mortality in the whole cohort were evidenced: an IAC, a Manheim peritonitis score ≥17, an American Society of Anesthesiology (ASA) score ≥3 and a SOFA score ≥1. The model’s Wald chi-squared statistic was 93.3 (df = 4, *P* <0.001). The Hosmer-Lemeshow test statistic was 3.8 (df = 6, *P* = 0.694). The model’s *c* statistic was 0.79 (0.73 to 0.85). For the population of patients with IAC (n = 69), 52.2% were treated with an antifungal drug (39% of azole and 61% of echinocandin). Mortality rate was 21.2% in the group treated versus 33.3% in the group not treated (*P* = 0.29).

**Table 7:** **Comparison of outcomes between yeast-positive and yeast-negative groups with complicated non-postoperative intra-abdominal infections**

**Whole cohort**	**Yeast positive**	**Yeast negative**	***P*** **value**
**(n = 442)**	**(n = 69)**	**(n = 373)**	
Any complication	54 (63.8)	158 (42.4)	0.001
Infectious complication	32 (46.3)	98 (26.3)	0.001
Digestive	8	33	
Pneumonia	12	27	
Miscellaneous	12	38	
Transfusion	19 (27.5)	38 (10.2)	0.001
Relaparotomy	17 (24.6)	52 (13.9)	0.02
Cardiovascular failure	36 (52.2)	64 (17.2)	<0.001
Respiratory failure	35 (50.7)	81 (21.7)	<0.001
ICU admission	39 (56.5)	111 (29.8)	<0.001
Duration of mechanical ventilation (d)	10.7 ± 14.9	9.5 ± 14.7	0.69
ICU length of stay (d)	16.1 ± 16.4	11.6 ± 13.9	0.11
Hospital length of stay (d)	20.5 ± 22.4	13.2 ± 16.0	0.001
Mortality	19 (27.5)	31 (8.3)	<0.001

Results are expressed as the mean ± standard deviation or the number (proportion, in %). ICU, intensive care unit.

**Table 8:** **Predictive factors of mortality in the whole cohort of 442 patients with complicated non-postoperative intra-abdominal infections**

**Parameters**	**Univariate analysis**			**Multivariate analysis**		
	**OR**	**95%CI**	***P*** **value**	**AOR**	**95%CI**	***P*** **value**
IAC	4.19	2.20-7.98	0.001	2.15	1.03-4.46	0.04
Ongoing AB ≥48 h	3.52	1.83-6.79	0.001	-	-	-
MPI score ≥17	7.96	3.31-19.10	0.001	3.22	1.26-8.25	0.02
ASA score ≥3	19.42	5.95-63.47	0.001	7.56	2.21-25.78	0.001
SOFA score ≥1	18.35	6.47-52.02	0.001	7.90	2.68-23.26	0.001
APACHE II score ≥7	19.22	5.88-62.82	0.001	-	-	-

OR, odds ratio; CI, confidence interval; AOR, adjusted odds ratio; IAC, intra-abdominal candidiasis; AB, antimicrobial therapy; MPI, Mannheim peritonitis index; ASA, American Society of Anesthesiology; SOFA, Sepsis-related Organ Failure Assessment; APACHE II, Acute Physiology and Chronic Health Evaluation II.

## Discussion

Our present results show that a YP peritoneal fluid culture is associated with worst outcomes and increased mortality in patients with CNPIAI. The prevalence of yeast isolation in this context is low (15.6%). Four parameters were independently associated with IAC: length of stay before surgery ≥48 h, per-operative cardiovascular failure, generalized peritonitis and upper gastrointestinal tract perforation. The predictive score has a high NPV and thus can be used to rule out the presence of yeast in the peritoneal fluid. Hence, this score may constitute an easy-to-use bedside tool that enables the physician to avoid the initiation of inappropriate antifungal treatment.

Although yeasts are undoubtedly pathogenic in postoperative infections [3], this question is subject to debate in the context of CAIs and there are few data on this specific topic. In a case-control study, there was no significant difference in outcome between YP and YN CAIs [3]. However, the study featured a small number of patients. In a study of patients with organ failure admitted to the ICU, a YP culture was found to be associated with elevated mortality [4]. Another study reported a significantly greater proportion of septic shock in CAIs when yeast was detected in the peritoneal fluid culture [23]. The overall prevalence of a YP culture in our study population (15.6%) is similar to the mean value reported in the literature (with values ranging from 4% to 43.4% in studies of CAIs) [2,24]. The prevalence clearly depends on the study population in question. For example, the prevalence was very high in two studies that focused solely on peptic ulcer perforations [1,24]. Furthermore, yeast isolation was associated with increased morbidity and mortality in these studies (21.7% vs. 3.4 % [1] and 33.3% vs. 14.6% [24] for YP and YN patients, respectively).

In the present study, four parameters were found to be independently associated with IAC of patients with a CNPIAI. Interestingly, upper gastrointestinal tract perforation and per-operative cardiovascular failure were previously included in a predictive score for severe intra-abdominal infections in the ICU [4]. Another Spanish study reported these two risk factors in 74 *Candida* peritonitis patients [25]. The two other parameters in the latter study (female gender and ongoing antimicrobial therapy for more than 48 hours) were not significant in our study [4]. However, ongoing antimicrobial therapy was associated with yeast isolation in our univariate analysis; this was probably due to the low prevalence of antimicrobial therapy because few patients had hospital-acquired infections. It has been well established that ongoing antimicrobial therapy is a recognized risk factor for candidemia [26]. However, in the present study, hospital length of stay ≥48 h before surgery was found to be independently associated with yeast isolation. There are no literature data on why yeast isolation is more frequently associated with generalized peritonitis than with localized infection. It could be only due to the major impact of appendicitis infections in CAI. The population investigated in the present study is mild to moderate with only a 33.9% rate of ICU admission and a low mortality of 11.3%. It is very different to the Dupont *et al.* [13] or Leon *et al*. [10] studies with a 100% rate of ICU admission and reported mortality of 43% and 33%, respectively.

The score developed in the present study has a good NPV. It may be important to avoid the inappropriate initiation of antifungal treatments that are costly and whose impact on resistance is not well known [27]. All the previously published scores or clinical rules were developed to predict the occurrence of candidemia (even in high-risk surgical patients) [9-11]. These scores have much the same operational values as our present score - notably with very high NPV for avoiding treatment. The high NPV is essentially due to the low prevalence of the disease. However, the predictive value of a score has been shown to be better for severe intra-abdominal infections in the ICU than previously described for candidemia, with a PPV of 67% and a NPV of 72% [13]. The colonization index and the *Candida* score were recently tested for the prediction of blood culture-negative IAC but had very poor operational values [28]. Blood levels of ß-glucan may be of value for the diagnosis of postoperative infections in high-risk surgical patients [28]. However, there are no data on the value of ß-glucan levels in CAIs. Furthermore, it may not be enough to know the change over time in ß-glucan levels in CAIs because the physician has to decide at bedside whether antifungal treatment must be initiated or not. We did not have access to a ß-glucan assay during our study. It has been suggested that a combination of high levels of ß-glucan and the *C. albicans* germ tube antibody can differentiate between *Candida* colonization and invasive candidiasis in ICU patients with severe abdominal conditions [29]. In the latter study, fewer than 50% of the patients had CAIs. Furthermore, the study was designed to assess the course of candidiasis in the ICU, rather than to predict the presence of candidiasis on admission.

A worse outcome associated with YP culture of the peritoneal fluid was evidenced in this study. Furthermore, it was independently associated with mortality. It is the first report in the literature of such impact in mild to moderate infections.

Our study had some potential limitations. First, this was a single-center study. Nevertheless, our center is a large tertiary-care hospital with an experienced, trained team for the care of patients with complicated intra-abdominal infections. Furthermore, peritoneal fluid samples were available for all patients and all were sent for microbiological and mycological culture. However, the present study’s results must be validated in multicenter trials. Recently, the sensitivity breakpoints for *Candida* spp. were modified according to the species [30]. This study used previously described breakpoints (>32 μg.ml^−1^) that could have underestimated the rate of strains resistant to fluconazole. Our study focused on the development of a predictive score and thus did not address the question of how best to treat IAC. It was only associated with increased morbidity and mortality in our population. The comparison of mortality rates according to treatment or not in the group of patients with IAC is not very relevant due to many confounding factors and a clear lack of power. The American guidelines for the treatment of abdominal candidiasis are essentially derived from candidemia guidelines [6]. Recently, an Italian group drew up a list of recommendations for the treatment of IAC [5]. However, in view of the lack of scientific evidence, the vast majority of guidelines are based on expert opinion or extrapolation of data on candidemia [8]. Lastly, the use of the new score is not well calibrated for critical care patients because only one-third of the cohort was admitted to the ICU.

## Conclusions

In conclusion, a score for predicting IAC was respectively built and validated in retrospective and prospective cohorts (featuring a total of 442 CNPIAIs). The new score has a high NPV (for ruling out the need for antifungal treatment at the bedside). It remains to be validated in larger, multicenter cohorts of patients.

## Key messages

- A simple clinical score at bedside may help to avoid antifungal treatment in patients with CNPIAIs.
- The clinical score includes per-operative cardiovascular failure (1 point), upper gastrointestinal tract location of the perforation (1 point), length of stay before surgery more than 48 hours (1 point) and generalized peritonitis (2 points)
- Yeast isolation in patients with CNPIAIs is associated with increased morbidity and independently with increased mortality.

## Abbreviations

APACHE II: Acute Physiology and Chronic Health Evaluation II
AUC: area under the curve
CAI: community-acquired infection
CI: confidence interval
CNPIAI: complicated non-postoperative intra-abdominal infection
IAC: intra-abdominal candidiasis
ICU: intensive care unit
NPNIAI: non-postoperative nosocomial intra-abdominal infection
NPV: negative predictive value
OR: odds ratio
PC: prospective cohort
PPV: positive predictive value
RC: retrospective cohort
ROC: receiver operating characteristics
SAPS II: Simplified Acute Physiology Score II
SD: standard deviation
SOFA: Sepsis-related Organ Failure Assessment
YN: yeast negative
YP: yeast positive
